# Deciphering the shared genetic structure between hip osteoarthritis and femoral neck bone mineral density

**DOI:** 10.3389/fgene.2025.1597005

**Published:** 2025-09-19

**Authors:** Jianguo Zhou, Junfu Na, Zongkun Jiang, Xiaoyan Dou, Shixuan Wang, Hongtao Li, Jian Kang

**Affiliations:** ^1^ Orthopedics and Traumatology, The Second Hospital of Liaoning University of Traditional Chinese Medicine, Shenyang, Liaoning, China; ^2^ Orthopedics and Traumatology, Affiliated Hospital of Liaoning University of Traditional Chinese Medicine, Shenyang, Liaoning, China; ^3^ Phase I Clinical Research Unit, Affiliated Hospital of Liaoning University of Traditional Chinese Medicine, Shenyang, Liaoning, China; ^4^ First Clinical College, Liaoning University of Traditional Chinese Medicine, Shenyang, Liaoning, China

**Keywords:** hip osteoarthritis, femoral neck bone mineral density, genetic loci, genetic heterogeneity, multi-tissue transcriptome-wide association studies

## Abstract

**Background:**

While the association between hip osteoarthritis (HOA) and femoral neck bone mineral density (FN-BMD) is established, their shared genetic architecture remains elusive. This study aims to explore the genetic correlation and underlying mechanisms.

**Methods:**

The present study applied bidirectional Mendelian randomisation (MR) to investigate causal relationships between HOA and FN-BMD. The quantification of genetic correlations was achieved by employing linkage disequilibrium score regression (LDSC) and high-definition likelihood (HDL) inference. Multi-trait analysis of genome-wide association studies (MTAG) have been shown to enhance statistical resolution, thereby unveiling hitherto unreported genetic associations. Independent MTAG-HOA risk loci were identified through conditional joint analysis (GCTA-COJO), complemented by functional mapping and annotation (FUMA) functional annotation. The application of both MAGMA and GCTA-fastBAT has revealed pleiotropic genes linked to MTAG-HOA susceptibility. Integration of fine-mapped genes from these loci with risk-associated candidates has enabled the identification of 13 key HOA-related genes. Functional annotation of these 13 key genes was performed using Gene Ontology (GO) and KEGG (Kyoto Encyclopedia of Genes and Genomes) pathway enrichment analyses. Multi-tissue transcriptome-wide association studies (TWAS) explored the expression of key genes across different tissues and their association with HOA. SMR analysis evaluated the causal relationship between key gene expressions in various tissues and HOA. Proteomic profiling is conducted via proteome-wide association studies (PWAS) and biomarker level imputation from summary statistics (BLISS). The application of stratified LDSC-SEG has revealed a genetic enrichment profile in cell types.

**Results:**

Bidirectional MR analysis revealed a significant negative causal effect of FN-BMD on HOA (β = −2.17, P < 0.01), whereas the reverse MR analysis did not identify a causal effect. LDSC and HDL analyses revealed genetic correlations between HOA and FN-BMD of rg = 0.132 and rg = 0.1697, respectively. GCTA-COJO and FUMA collectively identified 28 independent risk SNPs associated with HOA. MAGMA and GCTA-fastBAT identified 48 pleiotropic genes. Integrating independent risk loci and pleiotropic genes culminated in the identification of 13 key genes associated with HOA. An enrichment analysis revealed that 13 key genes were significantly associated with biological processes integral to cartilage development, osteogenesis, cell proliferation, apoptosis, and stem cell differentiation. Multi-tissue TWAS and SMR analyses indicated that seven genes were associated with HOA across 22 tissues, with brain tissues accounting for 28.6%. Furthermore, PWAS and BLISS methods were utilized to analyze the proteomic features of these key genes. LDSC-SEG analysis revealed enrichment of HOA heritability in Cartilage, Lymphocytes, Oocytes, B Lymphocytes, Germ Cells, Osteoblasts, and Embryoid Bodies.

**Conclusion:**

This study provides a comprehensive analysis of the genetic correlation between HOA and FN-BMD, elucidating shared genetic architecture and pinpointing key genes. These findings offer novel insights into the interplay between HOA and FN-BMD and highlight potential therapeutic targets.

## 1 Introduction

Osteoarthritis (OA) is a joint disease characterized by articular cartilage degeneration, subchondral bone remodeling and joint space narrowing (Motta et al., 2023). Its pathogenesis results from the intricate interplay between systemic predisposing factors and local biomechanical influences. Established risk factors reported include age, obesity, mechanical stress, metabolic disorders and genetic predisposition (Carpenter et al., 2006). Hip and knee joints, the primary weight-bearing joints in humans, are most affected by OA (Husted, 2012; Cui et al., 2020). Historically, OA has been regarded primarily as a disease of cartilage, with cartilage degradation as the central pathological feature. However, recent research has increasingly recognized the critical role of subchondral bone in the pathogenesis of OA. In hip osteoarthritis (HOA), femoral bone abnormalities manifest as both focal changes, including marginal osteophytes and subchondral bone cysts, and spatially heterogeneous changes, such as subchondral plate thickening and reduced trabecular bone mineralization (Chiba et al., 2014; Wang et al., 2016). Current understanding of changes in femoral bone density and microstructure in HOA focuses primarily on biomechanical stress distribution and biological responses (Auger et al., 2022). Nevertheless, the underlying mechanisms, particularly the role of genetic factors, remain to be fully elucidated.

It is estimated that the heritability of OA exceeds 50%. Previous genetic investigations have identified 27 risk loci across hip, knee, and hand osteoarthritis (Zengini et al., 2018), but locus overlap among these sites is limited. Mendelian randomization (MR) studies have evidenced a causal link between HOA and femoral neck bone mineral density (FN-BMD) (Qu et al., 2023). However, the genetic mechanisms underlying FN-BMD variation in HOA patients, and the extent of shared genetic architecture between HOA and FN-BMD, remain poorly understood. Genome-wide association studies (GWAS) have emerged as a robust tool for dissecting genetic risk factors in complex diseases. To address these knowledge gaps, the present study employs a suite of advanced post-GWAS analytical methodologies to systematically investigate the shared genetic architecture between HOA and FN-BMD, and to further identify potential pleiotropic genes. The overarching objective of this study is to comprehensively dissect the genetic architecture of FN-BMD variation in HOA, thereby providing a foundation for a deeper understanding of HOA pathogenesis and the development of targeted therapeutic interventions.

Research that focuses on just one disease might not identify the important genetic loci and molecular regulatory mechanisms. Therefore, multi-trait analytical approaches are essential for broadening the phenotypic spectrum under investigation, identifying associated risk loci and elucidating the shared genetic aetiology of different diseases (Lee et al., 2021). Shared genetic aetiology suggests potential pleiotropy, which often represents genetic confounders that link these traits (Sivakumaran et al., 2011; Verbanck et al., 2018). Consequently, cross-trait analyses leveraging correlations between GWAS data for HOA and FN-BMD are recommended to explore pleiotropic genetic variants or loci across multiple traits. These pleiotropic loci could be potential therapeutic targets, providing opportunities for simultaneous prevention or treatment of these conditions.

**FIGURE 1:**
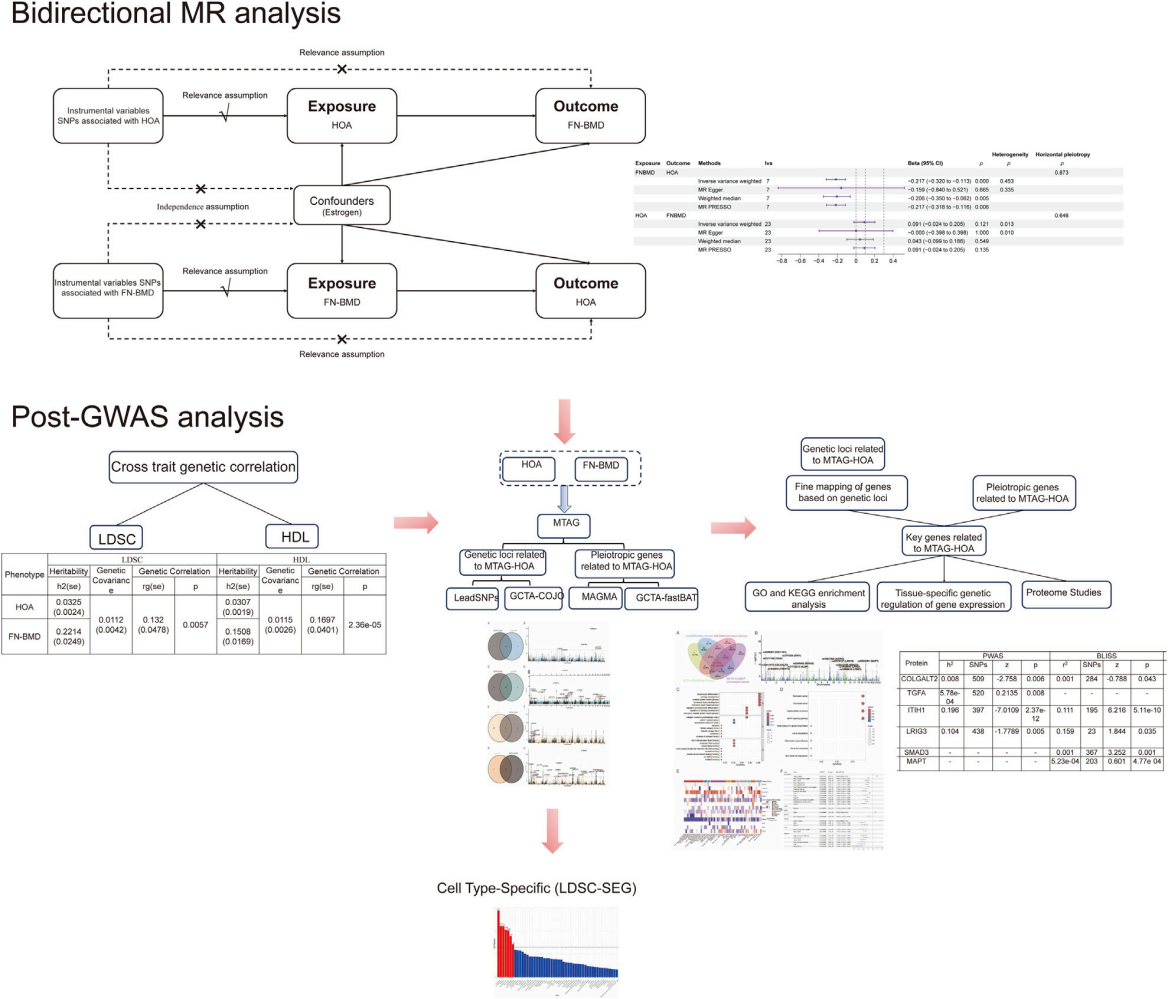
Flow chart of this study.

This study aimed to elucidate the genetic basis of HOA and FN-BMD, and to further investigate the biological mechanisms underlying HOA. Initially, bidirectional MR analysis was employed to assess the causal relationship between HOA and FN-BMD; genetic correlations were quantified using linkage disequilibrium score regression (LDSC) and high-definition likelihood (HDL) inference, and multi-trait analysis of GWAS (MTAG) was utilized to enhance statistical power and uncover novel genetic associations. Subsequently, independent HOA risk loci were identified and fine-mapped using conditional and joint analysis (GCTA-COJO) and FUMA (Functional Mapping and Annotation) for functional annotation. MAGMA and GCTA-fastBAT were then applied to identify pleiotropic genes associated with HOA susceptibility. Through the integration of genes mapped from risk loci and pleiotropic genes, 13 key HOA-associated genes were identified. Subsequently, functional annotation of these 13 key genes was performed using Gene Ontology (GO) and KEGG (Kyoto Encyclopedia of Genes and Genomes) pathway enrichment analyses. Following this, a multi-tissue Transcriptome-Wide Association Study (TWAS) was conducted to analyze the expression patterns of these key genes across various tissues and to assess their association with HOA. Summary-data-based SMR analysis was then used to further investigate the causal effects of gene expression in different tissues on HOA. Furthermore, the proteomic profiles of the 13 key genes were comprehensively analyzed using Proteome-Wide Association Study (PWAS) and the Biomarker Level Imputation from Summary Statistics (BLISS) method. Finally, stratified LDSC-SEG (LD Score Regression for specifically expressed genes) analysis was applied to identify cell-type-specific genetic enrichment, offering novel insights into the mechanisms of HOA (Figure 1).

## 2 Materials and methods

### 2.1 Data source

Summary statistics for HOA were obtained from a large GWAS available in the GWAS Catalog (GCST007091). This GWAS integrated genetic data from 393,837 individuals of European ancestry, including 15,704 HOA cases and 378,169 controls. Case samples were derived from the UK Biobank and the Arthritis Research UK Osteoarthritis Genetics Consortium (arcOGEN), with diagnoses based on clinical criteria (joint replacement) or radiographic assessment (Kellgren-Lawrence grade ≥ 2) (Tachmazidou et al., 2019).

Genetic variants associated with BMD were derived from the summary statistics of the largest publicly available GWAS meta-analysis conducted by the Genetic Factors for Osteoporosis Consortium (GEFOS). This dataset includes GWAS results for FN-BMD in 49,988 European ancestry, with BMD measurements obtained via dual-energy X-ray absorptiometry (DXA). The original GWAS analysis assessed the additive effects of single nucleotide polymorphisms (SNPs) with a minor allele frequency (MAF) > 0.5% on FN-BMD, accounting for key covariates such as sex, age, and body mass index (BMI). To facilitate downstream analyses, GEFOS applied weighting and standardization to the BMD phenotype data (mean = 0, standard deviation = 1) (Zheng et al., 2015).

### 2.2 Bidirectional MR analysis

MR analysis was employed to investigate the causal relationship between HOA and FN-BMD using SNPs as instrumental variables (IVs). The selection of IVs was based on fulfilling three core assumptions: 1 Relevance assumption: IVs must be strongly associated with the exposure; 2 Independence assumption: IVs must be independent of confounders; 3 Exclusion restriction assumption: IVs can only affect the outcome through the exposure and not through other pathways (Palmer et al., 2012).

The screening criteria for IVs included a strong association with exposure (p < 5 × 10^−8^), low linkage disequilibrium (LD) (r^2^ < 0.001), and F-values greater than 10 to mitigate weak instrument bias. Steiger filtering was applied to ensure the correct directionality of the causal effect. Outlier IVs were identified and excluded using the MR-PRESSO method. Potential confounders, such as estrogen, were identified and removed using the LDlink database (Lin et al., 2020). The primary MR analysis method used was Inverse Variance Weighted (IVW) analysis. Heterogeneity among IVs was assessed using Q-tests, and pleiotropy was evaluated using MR-Egger regression based on its intercept estimates.

### 2.3 Genetic evidence analysis

#### 2.3.1 Linkage disequilibrium score regression (LDSC)

LDSC is a statistical method used to estimate the genetic correlation (rg) between HOA and FN-BMD. LDSC evaluates the strength of the association between each SNP and the traits by estimating the LD score for each SNP. The results of cross-trait LDSC analyses are reported as genetic correlations and their standard errors (se). However, LDSC analysis may not provide reliable results if the heritability (h^2^) of either or both traits is low, which could limit the interpretation of the genetic correlation estimates (Bulik-Sullivan et al., 2015).

#### 2.3.2 High-definition likelihood inference (HDL)

HDL regression was employed to enhance the precision and robustness of genetic correlation estimates and to validate the findings from LDSC analysis. Compared to LDSC, HDL offers a more advanced approach to genetic correlation analysis, significantly improving estimation accuracy. By leveraging leading eigenvalues with greater explanatory power and eigenvectors of the LD score matrix, HDL more effectively captures genetic correlation signals. HDL regression reduces the variance in genetic correlation estimates by approximately 60%, thereby increasing statistical power comparable to a substantial expansion in sample size (Ning et al., 2020). The reference dataset for HDL analysis included an LD matrix and its eigenvalue decomposition derived from 335,265 UK Biobank participants, incorporating 1,029,876 high-quality HapMap3 SNPs.

### 2.4 Multi-trait analysis

MTAG is a method that employs generalized inverse-variance-weighted meta-analysis to integrate summary statistics from multiple GWAS datasets. This method has been extensively applied to investigate the shared genetic architecture among traits, and it is currently recognised as a principal approach for exploring the common genetic underpinnings of diverse phenotypes (Guo et al., 2022; Liu et al., 2024; Meng et al., 2025; Song et al., 2025). The primary objective of MTAG is to enhance the statistical power of GWAS and improve the detection of associated loci by capitalizing on genetic correlations among traits. While the primary objective of MTAG-derived GWAS results is to strengthen signals for the primary trait, loci associated with this primary trait that exhibits collinearity with other traits will also be evident. This will aid in the discovery of potential shared (pleiotropic) loci (Turley et al., 2018). Furthermore, as this study aims to elucidate the genetic architecture of HOA, GWAS data for HOA and FN-BMD were combined to construct MTAG datasets (MTAG-HOA and MTAG-FN-BMD), and subsequently, the MTAG-HOA dataset was selected for further analyses.

### 2.5 Identification of independent risk loci

#### 2.5.1 FUMA genetic association localization and annotation

The FUMA platform used stringent criteria with a maximum SNP P of < 5 × 10^−8^ and an additional inclusive significance threshold of P < 0.05. Independent SNPs were identified using an r^2^ threshold of < 0.6, with lead SNPs restricted to an r^2^ of < 0.1 within a 1 Mb radius. Genomic risk loci were defined by merged regions where lead SNPs were within 250 kb (Watanabe et al., 2017).

#### 2.5.2 Conditional and joint multiple-SNP analysis (COJO)

GCTA-COJO was conducted using GCTA software. COJO identified SNPs independently associated with MTAG-HOA susceptibility, reducing redundancy from multiple SNPs in high LD that might otherwise represent the same underlying signal. That is, any two loci with less than 1 Mb on the same chromosome were analyzed conditionally to observe whether other loci were also associated with the phenotype. If a SNP lost statistical significance after conditional analysis, its association with the phenotype was explained by other SNPs and was excluded from further analysis. The stepwise model selection framework implemented in GCTA-COJO was applied to perform conditional and joint association analysis (Yang et al., 2012). This approach identified significant, independently associated variants within established genomic risk loci (P.mtag < 5 × 10^−8^) and validated additional signals using a joint P threshold of < 5 × 10^−8^. This study used the 1000 Genomes Project Phase 3 European ancestry reference dataset (Auton et al., 2015).

### 2.6 Genetic insights into MTAG-HOA

#### 2.6.1 MAGMA analysis

MAGMA employs a multiple regression model to compute the cumulative effect of SNPs assigned to each gene (±10 kb). Subsequently, to quantify the gene-phenotype association, MAGMA aggregates SNP-level association statistics to derive gene scores (de Leeuw et al., 2015). The genome-wide Bonferroni-corrected significance level was set as P < 2.77 × 10^−6^ (0.05/18,079).

#### 2.6.2 GCTA-FastBAT analysis

GCTA-fastBAT framework implements efficient gene-based association testing through quadratic form aggregation of SNP-derived z-statistics within defined genomic regions. This set-based approach calculates P using the approximate distribution of χ^2^ statistic summation, with LD correction via the 1,000 Genomes Project Phase 3 reference panel (Bakshi et al., 2016). The genome-wide Bonferroni-corrected significance level was set as P_.FastBAT_ < 2.05 × 10^−6^ (0.05/24,390).

### 2.7 Functional enrichment analysis of MTAG-HOA-related key genes

Integration of findings from genes fine-mapped by GCTA-COJO and lead SNPs with those from pleiotropic genes identified through MAGMA and GCTA-fastBAT analyses consistently identified a specific set of genes as key determinants for MTAG-HOA. GO and KEGG databases were utilized for functional annotation, with a significance threshold of P < 0.05 applied. GO includes three domains: biological processes (BP), cellular components (CC), and molecular functions (MF) (Yu et al., 2012).

### 2.8 Multi-tissue transcriptome-wide association studie

The decision to incorporate all 49 tissues from GTEx v8 in the TWAS analysis was informed by the mounting recognition that skeletal health is not solely determined by local joint pathology, but also involves intricate interactions with the central nervous system (Zhang et al., 2025), peripheral nerves (Yoshino et al., 2021), digestive system (Liu et al., 2022), adipose tissue (Zapata-Linares et al., 2025), and immune responses (Xu et al., 2024). The GTEx v8 dataset provides comprehensive, high-quality expression reference panels across these major physiological systems, enabling tissue-specific evaluation of genetically regulated gene expressions (Li et al., 2018). Utilizing this comprehensive approach facilitates a more comprehensive investigation of the transcriptional architecture underlying MTAG-HOA and its genetic correlation with FN-BMD. By incorporating diverse tissue contexts, this study enhances the capacity to identify functionally relevant genes with context-dependent effects, thereby facilitating deeper insights into the multi-systemic etiology of hip osteoarthritis. MetaXcan leverages pre-established gene expression prediction models, which were trained within the GTEx v8 reference population and adjusted for sex, as well as potential technical and population-based confounding factors. These models facilitate the accurate estimation of the heritable component of gene expression levels in large, independent cohorts. The GTEx v8 prediction models are constructed upon the MASHR framework and, notably, do not simply incorporate all SNPs within a fixed window. Instead, they prioritize SNPs with a higher probability of causal involvement in gene expression regulation, using the DAP-G algorithm as a feature selection method (Wen et al., 2016). The DAP-G algorithm computes a posterior inclusion probability (PIP) for each SNP, retaining those with PIP >0.01 for model building (Urbut et al., 2019). This strategy is designed to improve both the predictive performance and biological interpretability of the models. As an extension of PrediXcan, MetaXcan integrates cis-eQTL weights derived from GTEx v8 and GWAS summary statistics, enabling analyses without requiring individual-level genotype data. This methodology presents a considerable advantage for the efficient identification of tissue-specific genes associated with traits of interest, particularly considering the growing availability of large-scale GWAS summary datasets. MetaXcan principally consists of two modules: S-PrediXcan and S-MultiXcan. S-PrediXcan was used to evaluate gene-trait associations within individual tissues, while S-MultiXcan was used to integrate association results across tissues (Barbeira et al., 2018; Barbeira et al., 2019). For all analyses, P-value were subjected to Benjamini-Hochberg (BH) correction, and a BH-adjusted P-value (P.adjust) < 0.05 was adopted as the threshold for statistical significance.

### 2.9 SMR analysis

SMR analysis leverages summary statistics from GWAS and eQTL to investigate potential pleiotropic associations between gene expression levels and complex traits. The Heterogeneity in Dependent Instruments (HEIDI) test is applied to evaluate potential horizontal pleiotropy within significant colocalization signals. The null hypothesis of the HEIDI test is the absence of horizontal pleiotropy within the colocalized signal. SMR and HEIDI methods together help determine whether alterations in gene expression levels mediate the SNP-phenotype effect, and to distinguish if this association reflects genuine causal mediation or is driven by linkage disequilibrium or horizontal pleiotropy (Zhu et al., 2016). This study employed SMR to investigate whether the expression levels of key genes mediate the genetic effects on MTAG-HOA, utilizing eQTL data from GTEx V8 as proxies for genetic variation. BH-adjusted P-value (P.adjust) < 0.05 was considered indicative of a significant SMR association. For the HEIDI test, P-value (P. HEIDI) > 0.01 suggests a lack of significant evidence for horizontal pleiotropy, thus supporting the interpretation that the SMR result potentially reflects a causally mediated effect of gene expression on the phenotype.

### 2.10 Proteomic studies

#### 2.10.1 PWAS analysis

PWAS were performed using FUSION with publicly available, pre-calculated, protein-level prediction models from the INTERVAL study (Sun et al., 2018). These models were derived from the INTERVAL study, in which the levels of plasma proteins were measured in 3,301 individuals of European ancestry using the SomaLogic platform. Subsequently, 3,222 predictive models were developed for 3,170 proteins. The PWAS analysis made use of European linkage disequilibrium (LD) reference data from the 1000 Genomes Project.

#### 2.10.2 BLISS analysis

BLISS is a novel method developed to create protein imputation models based on summary-level pQTL data, to conduct multi-ancestry PWAS analysis. By integrating proteomic data with GWAS findings, PWAS provides a more direct understanding of how genetic variations influence diseases through changes in protein abundance and function (Shan et al., 2025). In this study, drawing upon the deCODE study pQTL datasets, BLISS technology was employed to delineate the intricate proteomic signatures associated with MTAG-HOA.

### 2.11 LDSC-SEG

LDSC-SEG is an extension of stratified LD score regression (S-LDSC) designed to pinpoint relevant cell types or tissues for complex traits. The premise of LDSC-SEG is that if genomic regions associated with the heritability of a disease or trait are enriched for genes specifically expressed in a particular tissue, this tissue is likely to play a causal role in the disease etiology or trait biology. Utilizing gene expression profiles across 152 cell types curated by the Franke Lab as genomic annotations, LDSC-SEG assesses the expression levels of genes within these 152 cell types and ranks genes based on their cell-type specificity (Finucane et al., 2018).

## 3 Results

### 3.1 Bidirectional MR analysis

**FIGURE 2:**
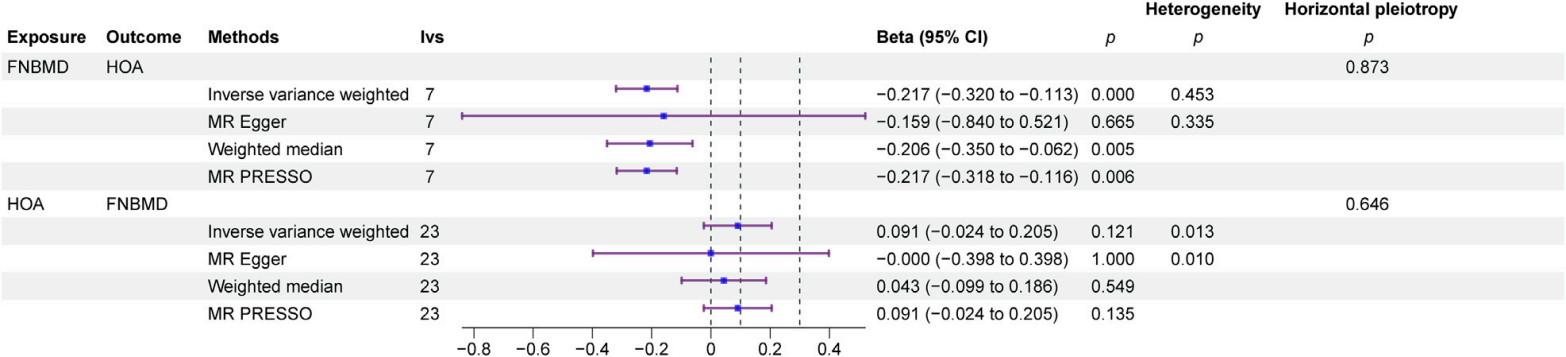
Bidirectional MR forest map.

MR analysis, employing IVs selected based on three core assumptions, F-statistics, and the Steiger filtering method, revealed a negative causal effect of FN-BMD on HOA (β = −2.17,P < 0.01). The Egger intercept analysis detected no significant horizontal pleiotropy (P > 0.1), suggesting that the observed causal effect was not biased by such pleiotropy. Cochran’s Q test revealed no statistically significant heterogeneity across instrumental variables (P > 0.05), indicating consistency in the causal estimates derived from different instruments. Conversely, reverse MR analysis indicated no causal effect of HOA on FN-BMD (β = −0.091,P = 0.121). The Egger intercept analysis for the reverse direction also detected no significant horizontal pleiotropy (P > 0.1), suggesting the absence of bias from such pleiotropy in this analysis. In the reverse MR analysis, Cochran’s Q test indicated significant heterogeneity (P < 0.05), suggesting variability in causal estimates across the different instrumental variables (Figure 2).

### 3.2 Genetic correlation between HOA and FN-BMD

Univariate LDSC analysis revealed that the h2 of HOA was 0.0325 (se = 0.0024), while that of FN-BMD was 0.2214 (se = 0.0249). Bivariate LDSC analysis further demonstrated a positive genetic correlation between HOA and FN-BMD (rg = 0.132, se = 0.0478, P = 0.0057).

HDL analysis revealed the heritability of HOA was 0.0307 (se = 0.0019), while FN-BMD exhibited the heritability of 0.1508 (se = 0.0169). In addition, there was a statistically significant positive genetic correlation between HOA and FN-BMD (rg = 0.1697, se = 0.0401, P = 2.36 × 10^−5^). Crucially, this positive genetic correlation robustly corroborates the findings from LDSC analysis, providing convergent evidence for a shared genetic architecture underlying HOA and FN-BMD (Table 1).

**TABLE 1:** Results of LDSC and HDL analyses.

Phenotype	LDSC				HDL			
	Heritability	Genetic covariance	Genetic correlation		Heritability	Genetic covariance	Genetic correlation	
	h2 (se)		rg (se)	p	h2 (se)		rg (se)	p
HOA	0.0325 (0.0024)	0.0112 (0.0042)	0.132 (0.0478)	0.0057	0.0307 (0.0019)	0.0115 (0.0026)	0.1697 (0.0401)	2.36e-05
FN-BMD	0.2214 (0.0249)				0.1508 (0.0169)			

Leveraging shared genetic signals between HOA and FN-BMD, MTAG boosts GWAS statistical power, resulting in an enhanced MTAG-HOA GWAS dataset. MTAG-HOA demonstrates a reduced false discovery rate (FDR) of 1.03%, indicating enhanced robustness of signal detection. Ultimately, the MTAG-HOA dataset comprises 6,673,816 validated SNPs, thereby conferring enhanced statistical power and reliability for subsequent analyses.

### 3.3 Risk loci for MTAG-HOA

**FIGURE 3:**
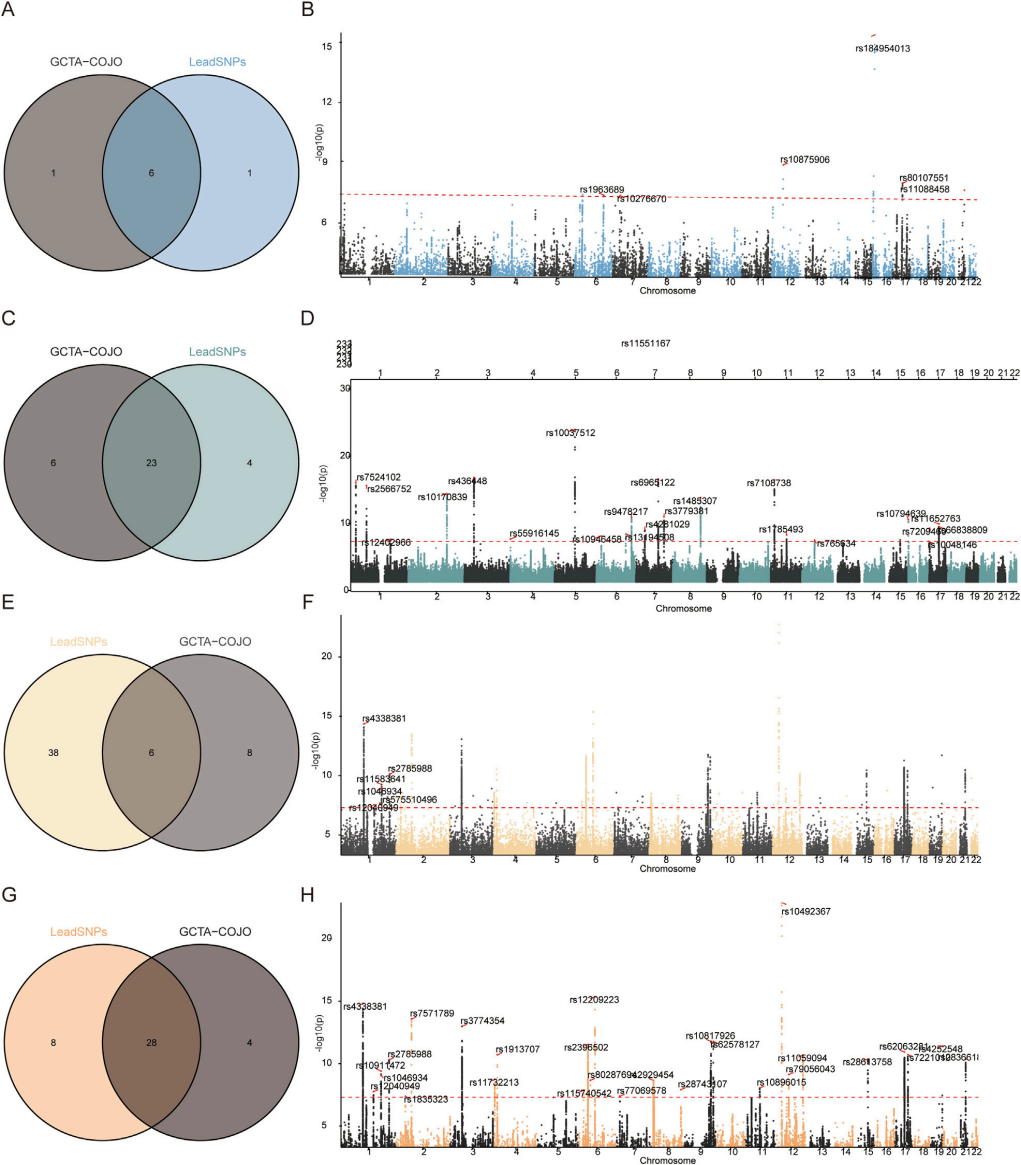
Venn diagram illustrating the intersection of GCTA-COJO and LeadSNP, and corresponding Manhattan plots with these risk loci annotated. **(A,B)** Venn diagram and Manhattan plot for FN-BMD. **(C,D)** Venn diagram and Manhattan plot for MTAG-FN-BMD. **(E,F)** Venn diagram and Manhattan plot for HOA; **(G,H)** Venn diagram and Manhattan plot for MTAG-HOA.

The FUMA platform was utilized to identify lead SNPs for GWAS-FN-BMD, GWAS-HOA, MTAG-FN-BMD, and MTAG-HOA. Initial analysis identified 7, 44, 27, and 36 lead SNPs, respectively, representing independent genomic loci associated with GWAS-FN-BMD, GWAS-HOA, MTAG-FN-BMD, and MTAG-HOA. To further refine these signals and dissect independent genetic contributions, GCTA-COJO analysis was applied to the summary statistics of GWAS-FN-BMD, GWAS-HOA, MTAG-FN-BMD, and MTAG-HOA. This analysis identified 7, 14, 29, and 32 SNPs, respectively, which collectively accounted for independent association signals. Cross-validation of the FUMA-derived lead SNPs and the GCTA-COJO-selected independent SNPs yielded a robust set of 6, 6, 23, and 28 variants, respectively. The present study concentrated principally on the independent genetic risk loci for MTAG-HOA. The 28 SNPs were thus designated as independent genetic risk variants for MTAG-HOA, thereby further underscoring the complex polygenic architecture of HOA and highlighting key genetic loci contributing to disease susceptibility (Figure 3; Supplementary Tables S1, S2).

### 3.4 Genetic insights into MTAG-HOA

MAGMA analysis identified 66 pleiotropic genes surpassing the multiple testing corrected P-value threshold (Supplementary Table S3). Independently, GCTA-fastBAT pinpointed 117 pleiotropic genes meeting the same significance criterion (Supplementary Table S4). Stringent cross-validation, by taking the intersection of gene lists from both methods, revealed a high-confidence set of 48 pleiotropic genes.

### 3.5 GO and KEGG analysis results

**FIGURE 4:**
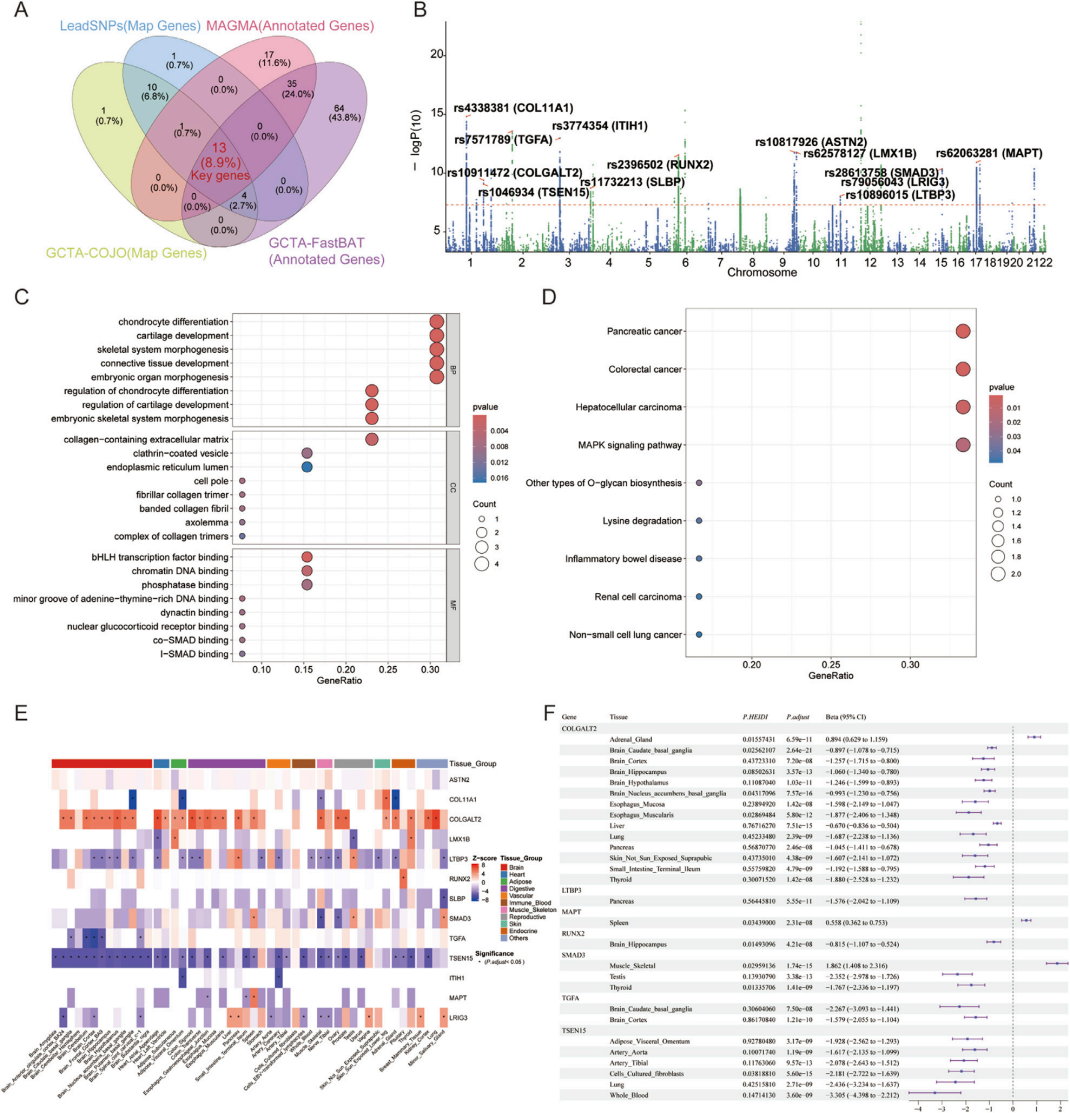
Functional enrichment and tissue-specific expression analyses of key genes. **(A)** Identification of key genes at the intersection of genes mapped from risk loci (co-identified by GCTA-COJO and LeadSNPs) and pleiotropic genes (identified by MAGMA and GCTA-fastBAT). **(B)** Manhattan plot illustrating the genomic distribution of key genes associated with MTAG-HOA. **(C,D)** Functional enrichment analysis (GO and KEGG) of the identified key genes. **(E)** Tissue-specific expression of the key genes across 49 tissues, based on TWAS. **(F)** Tissue-specific expression of the key genes across 49 tissues, based on SMR.

Integrating GCTA-COJO, FUMA, MAGMA, and GCTA-fastBAT analyses, this study identified 13 genes as potential key genes of MTAG-HOA (Figures 4A, B; Table 2). Among these risk loci, all are situated within intronic regions of key genes, except for rs1046934, which is in an exon of TSEN15. Notably, several of these gene loci, including those within RUNX2 (rs2396502), COL11A1 (rs4338381), LTBP3 (rs10896015), SLBP (rs11732213), LMX1B (rs62578127), LRIG3 (rs79056043), MAPT (rs62063281), and COLGALT2 (rs10911472), have been previously validated in independent studies as being intricately linked to the pathogenesis of HOA.

**TABLE 2:** Information on key genes and risk loci.

Risk loci	chr	pos	uniqID	Gene	func	MAGMA		GCTA-fastBAT	
						Z	P	SNPs	P
rs4338381	1	103572927	1p21.1	COL11A1	intronic	7.46	4.37e-14	116	5.09e-18
rs10911472	1	183908813	1q25.3	COLGALT2	intronic	5.44	2.67e-08	100	1.46e-13
rs1046934	1	184023529	1q25.3	TSEN15	exonic	5.16	1.22e-07	55	4.32e-08
rs7571789	2	70714793	2p13.3	TGFA	intronic	6.60	1.99e-11	140	6.35e-09
rs3774354	3	52817675	3p21.1	ITIH1	intronic	6.70	9.87e-12	31	1.15e-12
rs11732213	4	1704244	4p16.3	SLBP	intronic	5.32	4.99e-08	59	1.57e-07
rs2396502	6	45357699	6p21.1	RUNX2	intronic	6.63	1.58e-11	128	9.00e-12
rs10817926	9	119483436	9q33.1	ASTN2	intronic	6.36	9.76e-11	633	1.82e-07
rs62578127	9	129386860	9q33.3	LMX1B	intronic	5.74	4.65e-09	169	3.08e-10
rs10896015	11	65323725	11q13.1	LTBP3	intronic	4.91	4.33e-07	34	3.11e-07
rs79056043	12	59289598	12q14.1	LRIG3	intronic	5.73	4.79e-09	44	5.79e-09
rs28613758	15	67368659	15q22.33	SMAD3	intronic	5.51	1.79e-08	124	3.79e-08
rs62063281	17	44038785	17q21.31	MAPT	intronic	6.31	1.38e-10	81	1.44e-09

GO analysis revealed that the key genes were significantly enriched in biological processes associated with cartilage development, osteogenesis, cell proliferation, apoptosis, and stem cell differentiation. Notably, these genes play crucial roles in the differentiation of cells that form bone, the formation of bone tissue, and the process by which bone tissue is mineralized. The analysis further highlighted regulatory mechanisms, including the TGF-β signaling pathway and pathways governing stem cell proliferation and differentiation (Figure 4C). KEGG pathway analysis demonstrated enrichment of these genes in pathways such as the MAPK signaling pathway, pathways in cancer, and inflammatory responses. This finding suggests that these genes may have pivotal roles in tumorigenesis and immune reactions (Figure 4D).

### 3.6 Multi -tissue TWAS identifies MTAG-HOA relevant peripheral tissues

This study employed S-PrediXcan to investigate the expression profiles of these 13 genes across 49 tissues, with the false discovery rate controlled by multiple comparison correction. Following this, Z-scores were utilized in this study to assess the strength and direction of the gene expression-phenotype association. Results revealed that ASTN2 showed no significant expression in any of the 49 tissues examined. COL11A1 downregulation occurred in five tissues, including the Adrenal Gland and Brain Putamen basal ganglia, while COLGALT2 exhibited significant upregulation in 28 tissues such as the Lung and Heart Atrial Appendage. ITIH1 downregulation was detected in the Adipose Visceral Omentum and Artery Coronary. LMX1B exhibited downregulation in the Heart Atrial Appendage and Testis, while showing upregulation in Adipose Subcutaneous and Thyroid tissues. LRIG3 displayed downregulation in seven tissues (e.g., Muscle Skeletal and Brain Anterior cingulate cortex BA24), with concurrent upregulation detected in five tissues including Breast Mammary Tissue and Minor Salivary Gland. LTBP3 demonstrated downregulation in 18 tissues (spanning the Brain Frontal Cortex BA9 and Muscle Skeletal), whereas its upregulation was specifically observed in the Testis and Pancreas. MAPT showed downregulation in the Esophagus Gastroesophageal Junction and the Small Intestine Terminal Ileum, and significant upregulation in the Spleen. RUNX2 displayed marked upregulation in the Pituitary. SLBP demonstrated reduced expression in the Minor Salivary Gland. SMAD3 downregulated in the Ovary and Muscle Skeletal, whereas upregulation was observed in the Spleen, Testis and Minor Salivary Gland. TGFA revealed downregulation in five brain regions, including the Brain Cortex. Finally, TSEN15 levels were significantly downregulated in 13 brain tissues (e.g., Brain Cortex) and 20 other non-neural tissues (Figure 4E; Supplementary Table S5).

To enhance statistical power compared to single-tissue analyses, this study employed S-MultiXcan, integrating results from multiple single-tissue models to generate unified aggregated statistics. Following multiple testing correction, 13 gene-level associations identified by S-MultiXcan were evaluated. The results showed that ASTN2 did not reach the significance threshold in the S-MultiXcan association analysis, while the remaining 12 genes demonstrated statistically significant associations (Supplementary Table S6).

### 3.7 SMR analysis results

SMR analysis revealed that, among the 13 genes of interest, seven genes (COLGALT2, LTBP3, MAPT, RUNX2, SMAD3, TGFA, and TSEN15) met the pre-defined selection criteria (P.adjust < 0.05 and P. HEIDI > 0.01) across 49 tissues. Specifically, COLGALT2, SMAD3, and TSEN15 demonstrated significant associations across multiple tissues. LTBP3 showed a significant association in the Pancreas tissue. MAPT showed a significant association in the Spleen tissue. RUNX2 showed a significant association in the Brain Hippocampus tissue. TGFA showed significant associations in both the Brain Caudate basal ganglia and Brain Cortex tissues (Figure 4F; Supplementary Table S7).

### 3.8 Proteomic studies

Plasma proteomic models from individuals of European ancestry in the INTERVAL study were pre-calculated, and PWAS analysis was conducted to identify significant associations among the 13 key genes implicated by MTAG-HOA. The analysis identified four proteins (COLGALT2, TGFA, ITIH1, and LRIG3) with significant associations. Furthermore, BLISS analysis conducted with protein data from deCODE revealed significant associations for five proteins (COLGALT2, ITIH1, LRIG3, SMAD3, and MAPT) (Table 3).

**TABLE 3:** Proteome studies.

Protein	PWAS				BLISS			
	h^2^	SNPs	z	p	r^2^	SNPs	z	p
COLGALT2	0.008	509	−2.758	0.006	0.001	284	−0.788	0.043
TGFA	5.78e-04	520	0.2135	0.008	-	-	-	-
ITIH1	0.196	397	−7.0109	2.37e-12	0.111	195	6.216	5.11e-10
LRIG3	0.104	438	−1.7789	0.005	0.159	23	1.844	0.035
SMAD3	-	-	-	-	0.001	367	3.252	0.001
MAPT	-	-	-	-	5.23e-04	203	0.601	4.77e-04

### 3.9 LDSC-SEG analysis results

**FIGURE 5:**
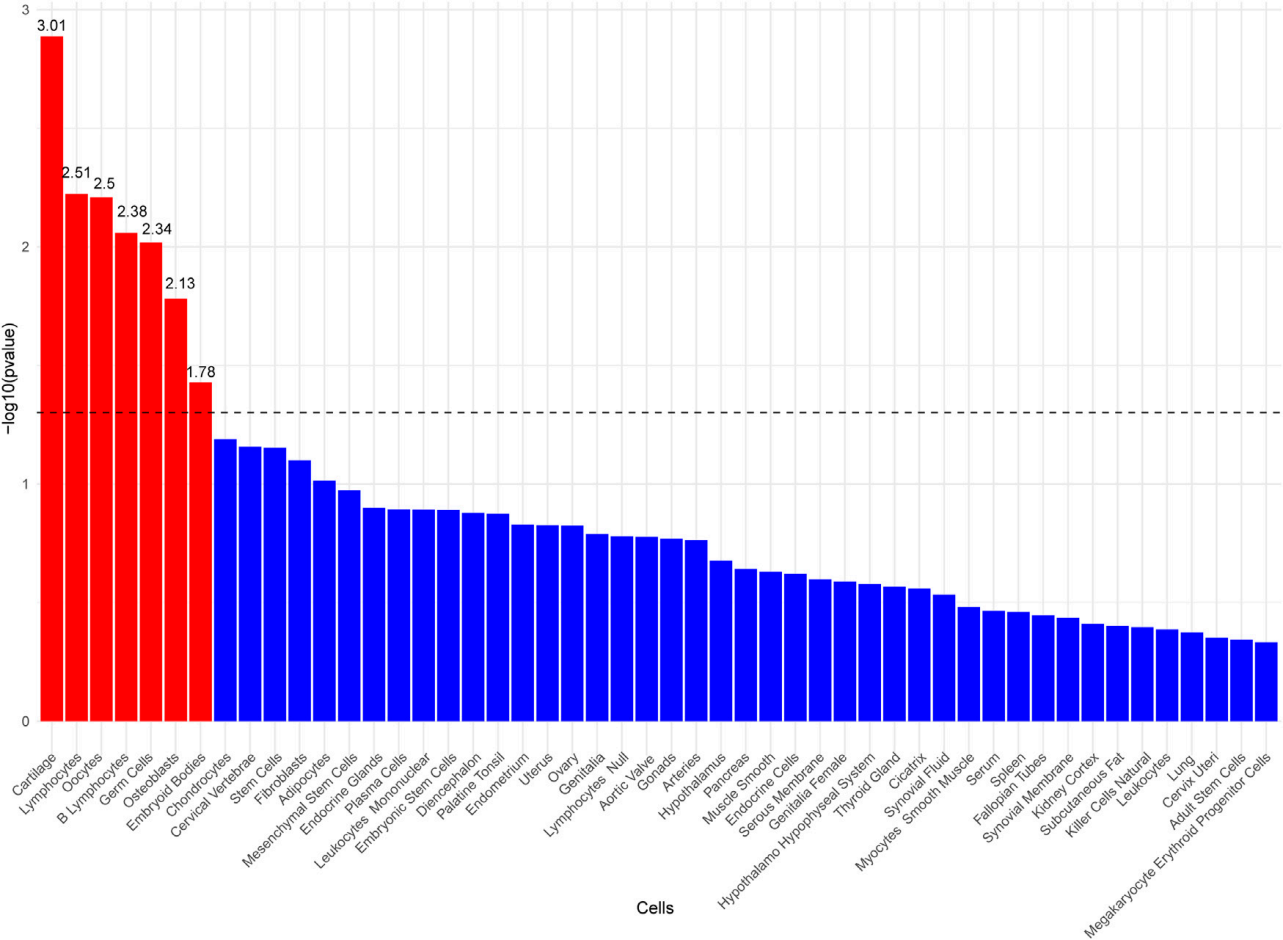
Bar plot of cell type-specific analysis using LDSC-SEG, displaying the top 50 enriched cell types ranked by p-value.

To investigate the potential cellular origins of MTAG-HOA, LDSC-SEG analysis was performed, referencing cell-type expression data from the Franke lab. The results indicated significant associations between MTAG-HOA and seven cell types—Cartilage, Lymphocytes, Oocytes, B Lymphocytes, Germ Cells, Osteoblasts, and Embryoid Bodies—at a coefficient level of P < 0.05 (Figure 5; Supplementary Table S8).

## 4 Discussion

### 4.1 Genetic correlation between HOA and FN-BMD

Emerging evidence over the past decade has implicated dynamic bone remodeling in the progression of OA, but the mechanistic links between BMD variations and joint degeneration phases remain poorly defined. Considering the pivotal role of genetic factors in both OA and its associated BMD changes, as well as their pleiotropic genetic architecture basis, traditional single disease approaches may prove insufficient to fully elucidate the underlying mechanisms. To address this gap, this study adopts a systematic and integrated strategy to comprehensively investigate the intricate genetic interactions driving the pathogenesis of both HOA and FN-BMD.

This study, employing bidirectional MR analysis, identified a negative causal effect of FN-BMD on HOA, whereas reverse MR analysis did not reveal such a causal relationship, this finding is consistent with previous MR studies (Qu et al., 2023). Furthermore, employing LDSC and HDL methods for global genetic correlation analysis, we elucidated the genetic correlation between HOA and FN-BMD, thereby providing robust evidence for their shared genetic architecture. Epidemiological studies have also indicated that osteophytic radiographic changes in HOA are associated with increased FN-BMD (Burger et al., 1996; Jadzic et al., 2022). Additionally, a longitudinal study demonstrated that radiographic changes characterized by joint space narrowing in HOA are also linked to increased BMD (Nevitt et al., 1995). The findings of this study are consistent with previous epidemiological studies, further strengthening these observations with robust genetic evidence derived from LDSC and HDL.

### 4.2 Risk loci, key genes and proteomics

To further increase the statistical power of the original HOA dataset, multi-trait analysis was applied to the GWAS datasets of HOA and FN-BMD. Analyses using GCTA-COJO and FUMA identified 28 independent risk loci significantly associated with MTAG-HOA, while MAGMA and GCTA-fastBAT analyses further identified 48 pleiotropic genes. Integrating the results of the GCTA-COJO, FUMA, MAGMA and GCTA-fastBAT analyses, the final refined set of 13 gene loci with significant association with MTAG-HOA was identified. These genes include COL11A1, COLGALT2, TSEN15, TGFA, ITIH1, SLBP, RUNX2, ASTN2, LMX1B, LTBP3, LRIG3, SMAD3, and MAPT. Subsequent proteomic analyses using PWAS and BLISS methods revealed key proteins significantly associated with HOA, including COLGALT2, TGFA, ITIH1, LRIG3, SMAD3, and MAPT. Integrating these findings underscores the potential importance of COLGALT2, ITIH1, and LRIG3 in HOA, and additionally reveals significant associations for TGFA, SMAD3 and MAPT. These findings not only deepen our understanding of the genetic mechanisms underlying HOA but also provide potential biomarkers and therapeutic targets for future research, thereby facilitating early diagnosis and precision treatment of HOA.

COLGALT2 encodes procollagen galactosyl transferase 2, an enzyme involved in the post-translational glycosylation of collagen (Schegg et al., 2009). Collagen is a fundamental structural and functional component of the extracellular matrix (ECM) in chondrocytes and its post-translational modifications are critical for its biological activity (Hardin et al., 2015). It has been demonstrated that increased glycosylation of collagen results in a reduction of intermolecular cross-linking, leading to a decrease in collagen fiber diameter and a concomitant reduction in tensile strength (Dominguez et al., 2005). Increased COLGALT2 expression, leading to elevated galactosyltransferase activity, is detrimental to cartilage health by impairing collagen biosynthesis (Rice et al., 2019). ITIH1 is synthesized by chondrocytes and interacts with hyaluronic acid and other extracellular matrix components, thereby conferring structural stability to cartilage (Zhao et al., 1995; Lord et al., 2020). Higher circulating ITIH1 levels have been associated with a reduced risk of HOA (Song et al., 2024). LRIG3 regulates neural crest development through modulation of the effector transcription factor Ets1 and modulates bone morphogenetic protein (BMP) signaling (Wang et al., 2015). Despite these established functions in neural development and signaling pathways, the exact role and underlying mechanisms of LRIG3 in the pathogenesis of OA remain to be thoroughly elucidated. SMAD3 maintains cartilage homeostasis and regulates inflammatory responses by activating the TGF-β signaling pathway and transducing signals from TGF-β receptors to the nucleus. Its mechanisms of action include mitochondrial dynamics, responses to mechanical stress and activation of proptosis. This signaling cascade is essential for chondrocyte function and the pathogenesis of OA (Liu et al., 2020). TGFA induces endothelin receptor A (ETA) expression in chondrocytes at both gene and protein levels, activating the RhoA/ROCK and MEK/ERK signaling pathways, ultimately driving cartilage degradation (Appleton et al., 2010). MAPT (microtubule-associated protein tau) is strongly associated with brain aging and neurodegenerative changes (Yi et al., 2025).

Although proteomic analysis revealed significant associations for the protein levels of six genes (COLGALT2, TGFA, ITIH1, LRIG3, SMAD3, and MAPT), other identified key genes did not exhibit such associations, potentially due to the limited comprehensiveness of current proteomic datasets. Consequently, despite the non-significant findings from proteomic analysis for these latter genes, their potential importance in the pathogenesis and progression of HOA should not be disregarded. RUNX2 is a transcription factor essential for skeletal system development and maturation, regulating physiological processes such as chondrocyte maturation and osteoblast differentiation (Komori, 2022). Studies have shown that RUNX2 overexpression promotes chondrocyte maturation and accelerates cartilage matrix degradation, contributing to the development of OA (Qin et al., 2015). Additionally, it facilitates the trans differentiation of terminal hypertrophic chondrocytes into osteoblasts in the femur (Takarada et al., 2013; Qin et al., 2020). Inhibition of COL11A1 results in impaired type II collagen fibril formation, subsequently affecting chondrocyte maturation and bone mineralization (Seegmiller et al., 1971). Moreover, COL11A1 suppresses lymphocyte enhancer-binding factor 1 (Lef-1), thereby inhibiting RUNX2-dependent transcriptional activation of the calcitonin promoter, ultimately affecting osteoblast differentiation and mineralization (Kahler and Westendorf, 2003). LTBP3 encodes latent TGF-β binding protein (LTBP) and plays a crucial role in TGF-β signaling regulation. Research indicates that TGF-β participates in particular chondrocyte formation and homeostatic regulation of cartilage matrix (Wang et al., 2018). Knockdown or loss of LTBP3 results in osteophyte formation, increased articular surface ossification, fibrosis, and cartilage loss, highlighting its critical role in skeletal homeostasis (Dabovic et al., 2002). LMX1B, a LIM homeobox domain transcription factor, regulates cell survival, proliferation, and inflammation. Inhibition of LMX1B enhances cell viability and suppresses chondrocyte apoptosis and inflammatory responses (Mu et al., 2022). SLBP, a cell cycle regulatory protein, facilitates the production of mature histones via 3′-end processing during the G1/S phase transition and is considered a critical gene significantly influencing joint health, particularly concerning risks associated with joint space width and hip cartilage thickness (Castaño-Betancourt et al., 2016; Wang et al., 2019). ASTN2 is highly expressed in both the developing and adult brain (Wilson et al., 2010). Studies have identified ASTN2 as a risk gene for HOA, however, its underlying mechanism remains unclear (Zeggini et al., 2012; Lindner et al., 2015). TSEN15 is a subunit of the tRNA splicing endonuclease complex. Although previous studies have indicated that TSEN15 is an independent risk gene for OA (Kehayova et al., 2023), its precise mechanisms of action remain to be further elucidated.

rs2396502, an intronic variant within the RUNX2 gene, has been identified by research as a significant risk locus for OA (Tachmazidou et al., 2019). Studies indicate that rs10896015, an intronic variant of the LTBP3 gene, is a functionally significant DNA variation significantly associated with HOA disease status at both genotype and allele levels (Zhao et al., 2020). rs10911472, an intronic variant within the COLGALT2 gene, has been suggested to influence COLGALT2 expression by modulating the methylation status of specific DNA methylation sites (cg18131582) in chondrocytes. This epigenetic regulatory mechanism may play a significant role in gene expression regulation, RNA splicing, and the generation of genetic diversity via alternative splicing (Aubourg et al., 2022). rs11732213, an intronic locus within the SLBP gene, has been demonstrated by research to have its epigenetic regulation in cartilage closely linked to SLBP function. SLBP is predominantly implicated in RNA splicing and gene expression regulation and is regarded as a pivotal gene that exerts a substantial influence on joint health, particularly about the risk associated with joint space width and hip cartilage thickness. (Castaño-Betancourt et al., 2016). The potential of rs11732213 to modulate the expression of SLBP and its associated genes by altering methylation patterns within cartilage is suggested. This regulatory activity could influence the onset and progression of HOA, thereby increasing susceptibility to the condition (Brumwell et al., 2022). rs62578127, an intronic variant within the LMX1B gene; rs4338381, an intronic variant within the COL11A1 gene; rs79056043, an intronic variant within the LRIG3 gene; and rs62063281, an intronic variant within the MAPT gene, are all considered significant risk loci for OA (Aubourg et al., 2022).

### 4.3 Multi-tissue analysis of potential gene expression

A multi-tissue analysis of potential gene expressions for MTAG-HOA was performed to identify and prioritize candidate causal genes and their relevant tissues. By integrating tissue-specific eQTL data from GTEx and employing TWAS (S-PrediXcan and S-MultiXcan) and SMR, the analysis identified eight gene expression regulatory loci associated with HOA across 22 tissues. Notably, brain tissue accounted for 28.6% of these associations, indicating significant enrichment. Cross-validation between transcriptome-wide association analysis and SMR highlighted potential causal associations of COLGALT2, RUNX2, and TGFA expression in brain tissue with HOA. Although the underlying mechanisms remain unclear, these findings suggest a potential role for the brain-bone axis in HOA pathogenesis, aligning with recent advancements in this emerging field. The brain-bone axis represents a complex bidirectional communication network linking the central nervous system and bone metabolism through diverse signaling pathways, neuroendocrine factors, and molecular mediators (Quiros-Gonzalez and Yadav, 2014). Growing evidence indicates that multiple neural pathways, including the sympathetic nervous system, hypothalamic neuropeptides, and neurotransmitters, play a crucial role in regulating both physiological and pathological bone changes (Karsenty and Ferron, 2012).

### 4.4 Enrichment analysis and cell specificity

This study employed GO and KEGG enrichment analyses, alongside LDSC-SEG cell-type specificity analysis, to thoroughly investigate the genetic relationship between HOA and FN-BMD and their underlying biological mechanisms. GO and KEGG enrichment analyses revealed that key genes were significantly enriched in pathways associated with biological processes crucial for skeletal development, cartilage formation, cell proliferation, apoptosis, and stem cell differentiation. These findings underscore the critical involvement of these genes in pivotal processes such as osteogenesis, bone tissue mineralization, and osteocyte differentiation, particularly within the TGF-β signaling pathway and mechanisms governing stem cell proliferation and differentiation. Concurrently, LDSC-SEG cell-type specificity analysis provided further insights into the cellular origins of HOA-associated genes. Integration with cell type-specific expression data revealed significant associations of MTAG-identified HOA loci with multiple cell types, including chondrocytes, osteoblasts, and immune cells such as lymphocytes and B lymphocytes. The associations with chondrocytes and osteoblasts particularly underscore the central role of these genes in bone tissue formation, whereas the relevance to immune cells suggests their potential pivotal involvement in immune responses and the inflammatory processes of osteoarthritis. These discoveries offer novel perspectives on the pathogenesis of HOA, particularly concerning how these genes function within different cell types and their interplay between the skeletal and immune systems.

### 4.5 Advantages and limitations

In summary, this study provides genetic evidence linking HOA and FN-BMD, while unraveling the genetic landscape of HOA. The strengths of this study are manifold. First, it integrates LDSC and HDL, leveraging the unique strengths of each method to minimize false-negative and false-positive findings. Second, the use of MTAG for multitrait GWAS analysis on the HOA and FN-BMD pathway represents a pioneering effort in this domain. Third, GCTA-COJO and FUMA revealed independent risk loci for MTAG-HOA, while MAGMA and GCTA-fastBAT identified 13 pleiotropic genes. Fourth, TWAS and SMR analyses identified eight genes whose expression regulation was associated with HOA risk across 22 tissues. Of note, brain tissue accounted for a substantial proportion of these associations.

It is imperative to acknowledge the limitations of this study. First, the focus on European populations necessitates cautious interpretation when extending conclusions to diverse racial and ethnic groups. Second, although TWAS and SMR analyses were conducted using the GTEx V8 eQTL dataset, only 49 tissues were included, and key tissues such as synovial tissue were not covered. Finally, although MTAG-HOA pleiotropic genes were identified, further longitudinal and experimental studies are crucial for elucidating the underlying biological mechanisms.

## 5 Conclusion

This study elucidates the genetic link between HOA and FN-BMD, highlights pleiotropic genes in HOA, and uncovers novel genetic risk factors. These findings deepen our understanding of HOA’s genetic landscape and may pave the way for developing innovative therapeutic strategies.

## Data Availability

The original contributions presented in the study are included in the article/Supplementary Material, further inquiries can be directed to the corresponding authors.

## Glossary

OA: Osteoarthritis
HOA: Hip Osteoarthritis
FN-BMD: Femoral Neck Bone Mineral Density
MR: Mendelian randomization
LDSC: Linkage Disequilibrium Score Regression
HDL: High-Definition Likelihood Inference
GWAS: Genome-Wide Association Studie
GCTA-COJO: conditional and joint analysis
FUMA: Functional Mapping and Annotation
GO: Gene Ontology
KEGG: Kyoto Encyclopedia of Genes and Genomes
TWAS: Transcriptome-Wide Association study
BMD: Bone Mineral Density
GEFOS: Genetic Factors for Osteoporosis Consortium
PWAS: Proteome-Wide Association Study
BLISS: Biomarker Level Imputation from Summary Statistics
SNP: Single-Nucleotide Polymorphism
se: Standard Error
MTAG: Multi-trait analysis of GWAS
DXA: dual-energy X-ray absorptiometry
MAF: minor allele frequency
BMI: body mass index
IV: instrumental variables
IVW: Inverse Variance Weighted
BP: biological processes
CC: cellular components
MF: molecular functions
BH: Benjamini-Hochberg
FDR: False Discovery Rate
SMR: summary-data-based MR
S-LDSC: Stratified LD Score Regression
Lef-1: Lymphocyte Enhancer-Binding Factor 1
ECM: Extracellular Matrix
ETA: Endothelin Receptor A
BMP: Bone Morphogenetic Protein
